# Revealing the epigenetic effect of temozolomide on glioblastoma cell lines in therapeutic conditions

**DOI:** 10.1371/journal.pone.0229534

**Published:** 2020-02-26

**Authors:** Agnieszka Belter, Jakub Barciszewski, Anna-Maria Barciszewska

**Affiliations:** 1 Institute of Bioorganic Chemistry, Polish Academy of Sciences, Poznan, Poland; 2 Intraoperative Imaging Unit, Chair and Department of Neurosurgery and Neurotraumatology, Karol Marcinkowski University of Medical Sciences, Poznan, Poland; 3 Department of Neurosurgery and Neurotraumatology, Heliodor Swiecicki Clinical Hospital, Poznan, Poland; Sechenov First Medical University, RUSSIAN FEDERATION

## Abstract

Temozolomide (TMZ) is a drug of choice in glioblastoma treatment. Its therapeutic applications expand also beyond high grade gliomas. However, a significant number of recurrences and resistance to the drug is observed. The key factor in each chemotherapy is to achieve the therapeutic doses of a drug at the pathologic site. Nonetheless, the rate of temozolomide penetration from blood to cerebrospinal fluid is only 20–30%, and even smaller into brain intestinum. That makes a challenge for the therapeutic regimens to obtain effective drug concentrations with minimal toxicity and minor side effects. The aim of our research was to explore a novel epigenetic mechanism of temozolomide action in therapeutic conditions. We analyzed the epigenetic effects of TMZ influence on different glioblastoma cell lines in therapeutically achieved TMZ concentrations through total changes of the level of 5-methylcytosine in DNA, the main epigenetic marker. That was done with classical approach of radioactive nucleotide post-labelling and separation on thin-layer chromatography. In the range of therapeutically achieved temozolomide concentrations we observed total DNA hypomethylation. The significant hypermethylating effect was visible after reaching TMZ concentrations of 10–50 μM (depending on the cell line). Longer exposure time promoted DNA hypomethylation. The demethylated state of the glioblastoma cell lines was overcome by repeated TMZ applications, where dose-dependent increase in DNA 5-methylcytosine contents was observed. Those effects were not seen in non-cancerous cell line. The increase of DNA methylation resulting in global gene silencing and consecutive down regulation of gene expression after TMZ treatment may explain better glioblastoma patients’ survival.

## Introduction

The therapeutic potential of temozolomide (TMZ) in the treatment of primary and recurrent glioblastoma (GBM) has been proven [1,2]. Its therapeutic applications expand also beyond high grade gliomas [3–5]. TMZ is most effective in glioblastomas with hypermethylated promotor of the *O*^*6*^*-methylguanine-DNA methyltransferase* (*MGMT*) [1,6–8]. However, the questions about its efficacy, patient selection, outcome, and prognosis still remain, and therapy failures are observed in the vast majority of glioblastoma patients. The changing of dosing regimens didn’t fulfill the expectations to increase the treatment effectivity [9].

**Fig 1:**
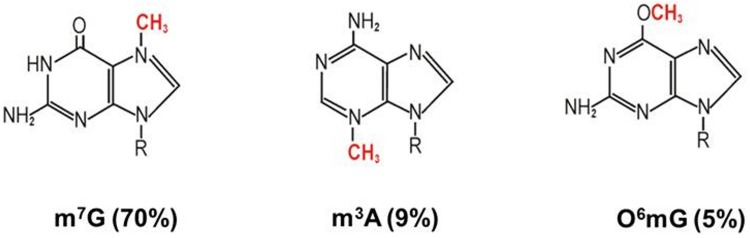
The main reaction products of the TMZ reaction with DNA. 7-methylguanosine (m^7^G) is the most abundant product (60–80%) of TMZ reaction with DNA, followed by 3-methyladenosine (m^3^A, 10–20%) and O6-methylguanosine (O^6^mG, 5%). Other products constitute ca. 15% [17].

Temozolomide (4-methyl-5-oxo-2,3,4,6,8-pentazabicyclo [4.3.0] nona-2,7,9-triene-9-carboxamide) is a small prodrug, with a molecular weight of 194.15, that undergoes chemical conversion at physiological pH to the active species 5-(3-methyl-1-triazeno)imidazole-4-carboxamide (MTIC), and generates an active methyl group, that reacts with DNA bases [10]. The cytotoxic activity of TMZ manifests mainly through methylation at the O^6^ position of guanine. Although that methyl adduct comprises less than 5% of the total temozolomide induced DNA modifications [11–13], O^6^-methylguanine leads to DNA mismatch repair, which results in preservation of the lesion and double-strand breaks, what leads to cell apoptosis [14,15]. Other alkyl adducts, like N^7^-methylguanine and N^3^-methyladenine, comprising ca. 80% of the total TMZ methylation products, are generally not cytotoxic, while they are easily repaired by the base excision repair system [12,13] (Fig 1). Therefore it is believed that generation of O^6^-methylguanine and a functional DNA mismatch repair pathway are both critical to the cytotoxic potential of temozolomide. Consequently, the currently accepted mechanism of resistance to TMZ is the activity of O^6^-methylguanine DNA methyltransferase (MGMT), the DNA repair enzyme [6]. However, in the light of numerous TMZ effects in the cell, cannot be the only one [16].

The site of TMZ action (brain) requires its effective entrance into the central nervous system (CNS) through the blood brain barrier (BBB). That is achieved with the unique chemical structure as well as pharmacokinetic properties [18,19]. Moreover, with oral administration, 100% of the given TMZ dose gets into blood flow [20]. However, the successful chemotherapy requires effective drug concentration in the pathologic site.

The studies on TMZ penetration into CNS done in non-human primate models have shown that the peak levels of the drug were: 104±3 μM in plasma (0.5 hrs after infusion end), and 26±4 μM in the cerebrospinal fluid (CSF) (2.5 hrs after infusion end), therefore the CSF:plasma ratio was 0.33±0.06 [19]. League-Pascual et al. observed that the degrees of CSF drug penetration after intranasal and intravenous administration were 36(32–57) and 22(20–41) %, respectively. The maximum TMZ drug concentration in the CSF was lower after intranasal delivery compared to intravenous administration due to the lower dose administered [21]. In the pharmacokinetic study on TMZ penetration in humans, drug concentration in the CSF was 20% of that of plasma, with an increase of 15% in case of concomitant radiochemotherapy. However, the active metabolite, MTIC, does not effectively penetrate the CNS [22]. With microdialysis method performed in GBM patients an average brain intestinum:plasma ratio was estimated as 17.8%. The mean peak TMZ concentration in brain tissue was 0.6±0.3μg/ml, and the mean time to reach peak level in the brain was 2.0±0.8 hrs [23].

Recently we showed that temozolomide modulates 5-methylcytosine (m^5^C), the main epigenetic marker, contents in cancer cells’ DNA [17]. An increasing TMZ concentration in the short time induced an increase of total m^5^C content in DNA (hypermethylation), but at extremely high (1000 μM) TMZ concentration and longer exposition time DNA hypomethylation was observed. DNA hypermethylation can be only explained by activation of DNA methyltransferase (DNMT), because TMZ is not a substrate for DNMT [24]. The other TMZ effect on the cell is chemical and oxidative stress. In such conditions 5-methylcytosine is a target for oxidation, what results with the decrease (demethylation, hypomethylation) of its total genomic contents [25]. Therefore changes in DNA methylation provide an appropriate picture of the disease [26]. In our previous studies we have also shown that m^5^C is a good marker for the diagnosis of brain tumors, hypertension, and other diseases [27–29]. However, the concentrations of TMZ used (100–1000 μM) in our previous study [17] didn’t correspond well with those reached in regular therapy in brain tissue and cerebrospinal fluid [19,21–23]. Therefore in the present analysis we decided to focus on the concentrations potentially achieved in the brain during TMZ therapy (mean 3 μM±1.5 μM [23]).

The aim of the current project was to evaluate the epigenetic influence of temozolomide treatment explaining its effects in glioblastoma, and other pathologies, in therapeutic concentrations and mimicking the therapeutic schemata.

## Materials and methods

### Chemicals

Temozolomide (Sigma-Aldrich/Merck) was freshly dissolved in dimethyl sulfoxide (DMSO, Sigma-Aldrich/Merck) at a concentration of 0.103 M. TMZ stock solution was used to prepare the required concentration with the complete medium.

### Cell lines’ source and identification

Human glioblastoma cell lines (T98G, U138, U118) and human keratinocyte (non-neoplastic) cell line (HaCaT) were purchased from ATCC (American Tissue Culture Collection, US; Cat. No. ATCC^®^ CRL-1690^™^, ATCC^®^ HTB-16^™^, ATCC^®^ HTB-15^™^, and ATCC^®^ CRL-2404^™^ respectively). ATCC’s recommended basic benchmark verification tests were implemented.

### Cell culture conditions

T98G and U138 cell lines were cultured in EMEM medium (ATCC), U118 in DMEM (ATCC), HaCaT in EMEM (Sigma-Aldrich/Merck). Each medium was supplemented with 10% (v/v) fetal bovine serum (FBS, Gibco) and antibiotics (ATCC, penicillin 10 U/ml, streptomycin 10 μg/ml, amphotericin B 25 ng/ml). Cells were cultured at 37°C with 5% CO_2_ in humidified air. Cell lines were seeded at a density of 5x10^5^ cells per well in 6-well plates containing 1 ml appropriated medium. Cells with 90–95% confluence, after ~24 hrs, were washed with phosphate-buffered saline (PBS, Sigma-Aldrich/Merck), placed in fresh medium and underwent treatment with TMZ as described below.

Safety procedures were used with cell lines to avoid contamination from other cell lines and microbes. Low-passage (up to 25) cell lines were used. Plasmocin^™^ Prophylactic (InvivoGen), a broad-spectrum anti-mycoplasma reagent, was used at 5 μg/ml in liquid media to prevent mycoplasma contamination. It is also active on a broad range of Gram-positive bacteria, such as *Staphylococcus* species, and Gram-negative bacteria. Medium with fresh Plasmocin was changed every 3 days. Plasmocin^™^ Prophylactic (5 μg/ml) exhibits no toxicity in eukaryotic cells. Cell cultures were monitored with PlasmoTest^™^ (InvivoGen). Mycoplasma was not detected in our cell cultures.

### Cell lines’ treatment with TMZ

The TMZ stock solution was added directly to culture medium according to designed concentrations:

1. TMZ at 0.5, 1, 3, 5, 10, 20, 30, 50 and 100 μM for 3, 12, 24 and 48 hrs.
2. TMZ at 0.5, 1, 3, 5, 10 μM for 5 days, wherein after 24, 48, 72 and 96 hrs cells were washed with PBS and placed in fresh medium with indicated TMZ concentrations.
3. TMZ at 0.5, 1, 3, 5, 10 μM for 24, 48, 72, 96, 120 and 168 hrs.

The control cells were treated with H_2_O or DMSO only. After appropriated (indicated above) time with TMZ, the cells were washed with PBS, trypsinized and collected by centrifugation at 3500 rpm for 5 min. The cellular pellets were quickly frozen and stored at—20°C for DNA isolation.

MTT assay for TMZ was carried out during our previous experiments [17]. It was shown that TMZ treatment in the range of 1–2000 μM triggers cell death apoptosis independent.

### DNA isolation from cell cultures

DNA from tissue samples was extracted with Genomic Mini kit (A&A Biotechnology, Poland). Shortly, tissue samples were incubated with RNase A, and then with proteinase K. After centrifugation (15000 rpm for 3 min), the supernatant was applied to mini column and DNA bound to the column was eluted with Tris-buffer pH 8.5 and stored at -20°C for further analysis. The purity and concentration of DNA preparations was checked by measuring of UV absorbance at 260 and 280 nm. The A_260_/A_280_ ratio was 2.0–2.1.

### DNA hydrolysis, labelling and TLC chromatography

**Fig 2:**
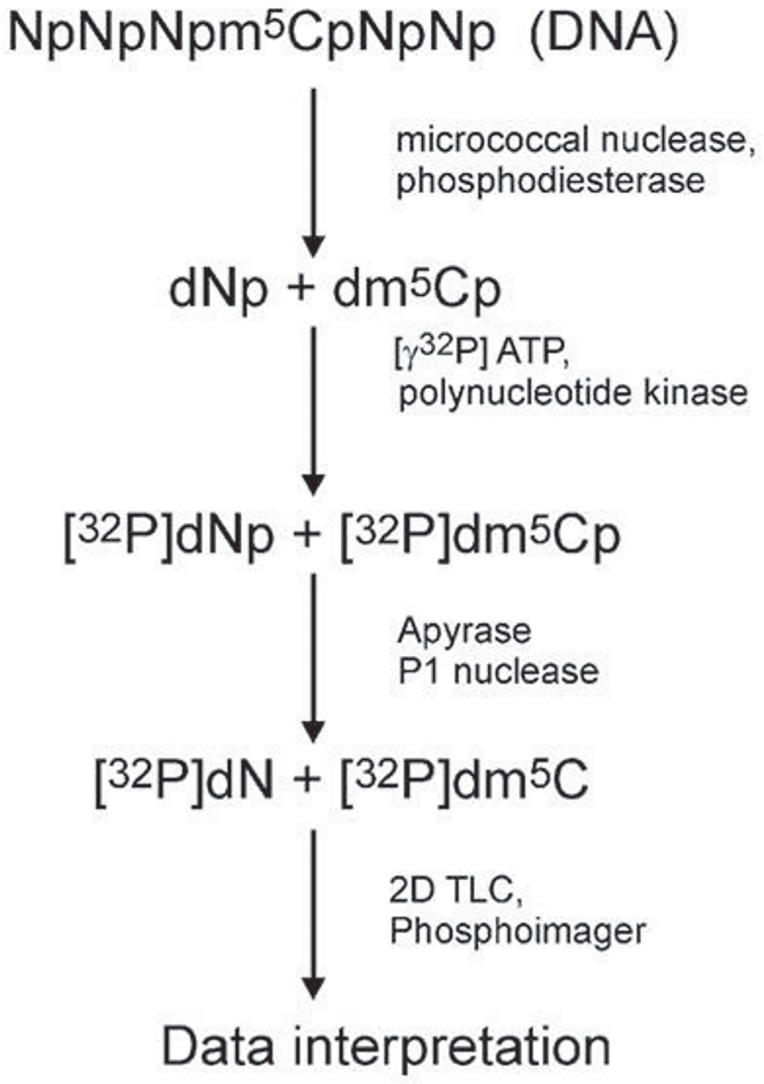
A flow chart of the total DNA methylation (m^5^C) analysis. Isolated DNA is hydrolyzed to 3’-mononucleotides (Np: A/adenosine, G/guanosine, C/cytidine, T/thymidine). The hydrolysate is labelled with [γ-^32^P] ATP, dephosphorylated (detachment of 3’ phosphate) and separated with two-dimensional TLC. The chromatogram is then evaluated with phosphoimager and the spots’ intensities are measured. Those values are used for the calculation of the R coefficient according to the given equation [27].

1 μg of dried DNA was dissolved in a succinate buffer (pH 6.0) containing 10 mM CaCl_2_ and digested with 0.001 units of spleen phosphodiesterase II and 0.02 units of micrococcal nuclease in 3.5 μl total volume for 5 h at 37°C. 0.17 μg of DNA digest was labeled with 1 μCi [γ-^32^P]ATP (6000 Ci/mM; Hartmann Analytic GmbH) and 1.5 units of T4 polynucleotide kinase in 3 μl of 10 mM bicine-NaOH pH 9.7 buffer containing 10 mM MgCl_2_, 10 mM DTT, and 1 mM spermidine. After 0.5 h at 37°C 3 μl of apyrase (10 units/ml) in the same buffer were added and incubated for another 0.5 h. The 3’ nucleotide phosphate (from [^32^P]dNp) was cleaved off with 0.2 μg RNase P1 in 500 mM ammonium acetate buffer, pH 4.5. Identification of [^32^P]dN was performed with a two-dimensional thin-layer chromatography on cellulose plates (Merck, Germany) using solvent system: isobutyric acid:NH_4_OH:H_2_O (66:1:17 v/v) in the first dimension and 0.1 M sodium phosphate (pH 6.8)-ammonium sulfate-*n*-propyl alcohol (100 ml/60 g/1.5 ml) in the second dimension. Radioactive spot analysis was done with the Phosphoimager Typhoon Screen (Pharmacia, Sweden) and ImageQuant Software (GE Healthcare, USA). For precise calculation we used the amount of material in spots corresponding to m^5^dC (5-methylcytosine), dC (cytosine) and dT (thymine). The total m^5^C contents was calculated as R = (m^5^dC/(m^5^dC+dC+dT))×100 (Fig 2).

### Statistical analysis

Microsoft Excel 2010 software with Data Analysis package was used for statistical analysis of all data. The data are the result of three independent experiments. Descriptive statistics function was used to generate mean and SD (S1–S3 Tables). One-tailed t-test was used to calculate significant differences of R values for tested samples as compared with control in experiments 1–3.

## Results

We analyzed changes in 5-methylcytosine (epigenetic marker) contents in DNA of three types of human glioblastoma cell lines (T98G, U138, U118), and one human keratinocyte cell line (HaCaT) after treatment with 0.5–100 μM of TMZ in the time range between 3 hrs and 7 days in different treatment regimens. These TMZ concentrations were chosen to cover the possible concentration of TMZ in the brain during chemotherapy (0.6±0.3μg/ml, corresponding to 3±1.5 μM) [19,21–23], and perform the observation after reaching the peak concentration in the brain (2.0±0.8 hrs) [23], as well as standard chemotherapy course time [1].

**Fig 3:**
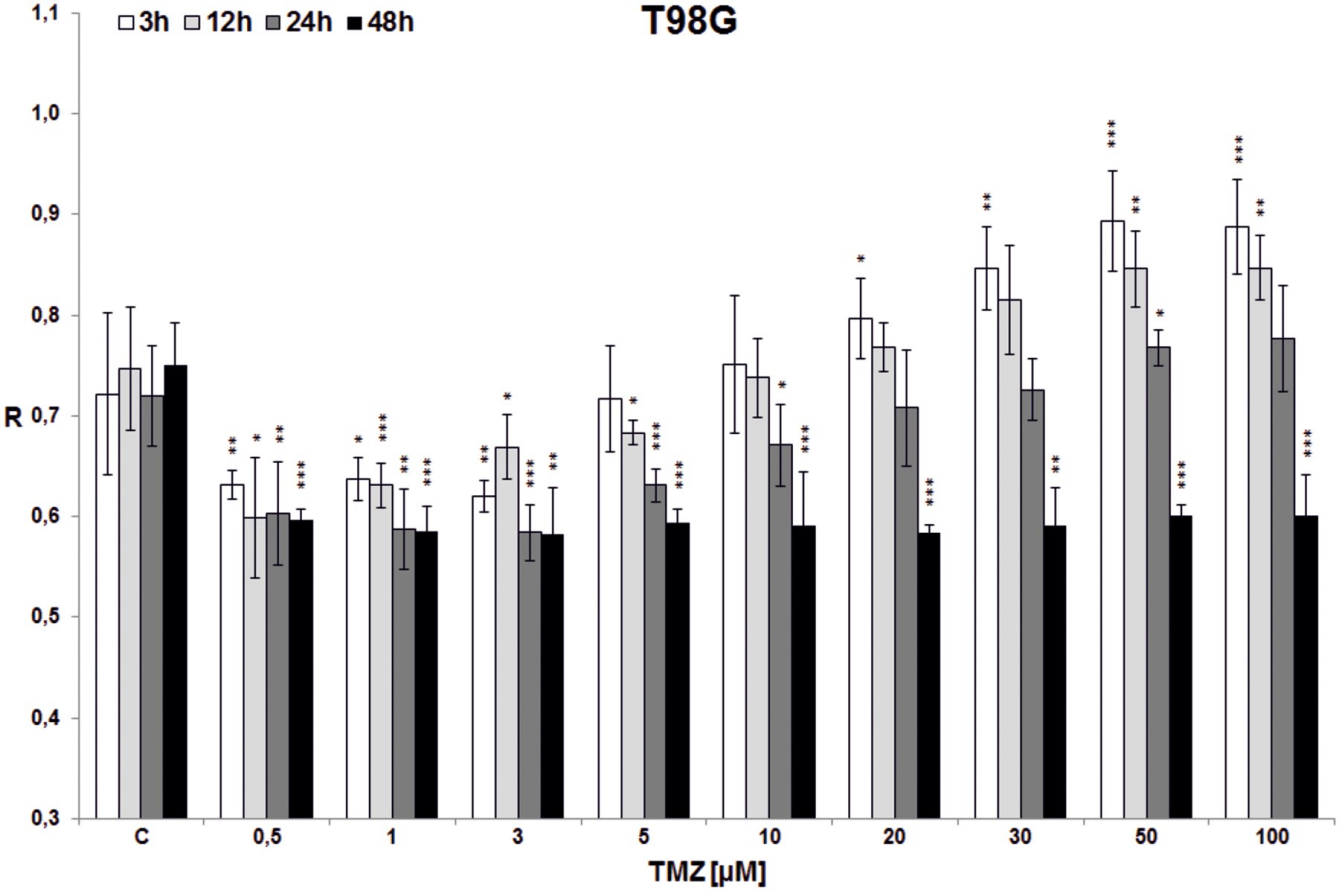
The effect of TMZ on total DNA methylation of T98G cell line. The analysis was performed after 3 (white bars), 12 (light grey bars), 24 (dark grey bars) and 48 (black bars) hrs of incubation in a given TMZ concentration (0.5–100 μM). Control cells (C) were treated with DMSO only. The R values are means from three experiments ±SD. Asterisks indicate a significant difference (*p < 0,05, **p < 0,01, ***p < 0,001) from the control (DMSO) value. Striking DNA hypomethylation happens in therapeutically achieved concentrations (less than 5 μM). Significant DNA hypermethylation is achieved at minimal TMZ concentrations of 20–50 μM in 3–24 hrs incubation time. Clear demethylating tendency is seen with 48 hrs incubation time in all TMZ concentrations.

**Fig 4:**
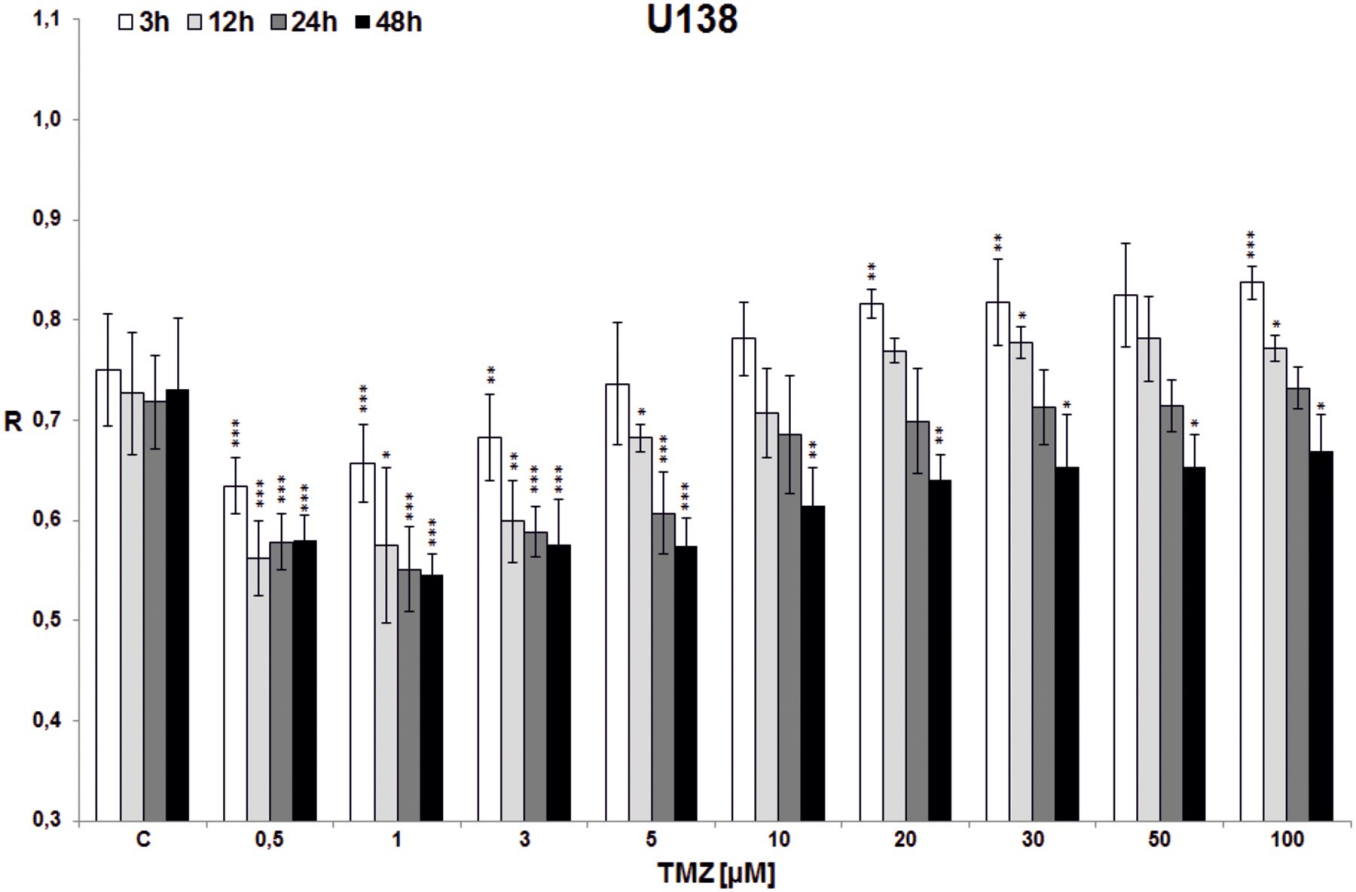
The effect of TMZ on total DNA methylation of U138 cell line. The analysis was performed after 3 (white bars), 12 (light grey bars), 24 (dark grey bars) and 48 (black bars) hrs of incubation in a given TMZ concentration (0.5–100 μM). Control cells (C) were treated with DMSO only. The R values are means from three experiments ±SD. Asterisks indicate a significant difference (*p < 0,05, **p < 0,01, ***p < 0,001) from the control (DMSO) value. A very little DNA hypermethylating effect is observed (for incubation times 3 and 12 hrs, over concentrations 20 and 30 μM respectively). Very clear initial hypomethylation is seen in TMZ concentrations up to 100 μM for incubation times of 24 and 48 hrs.

**Fig 5:**
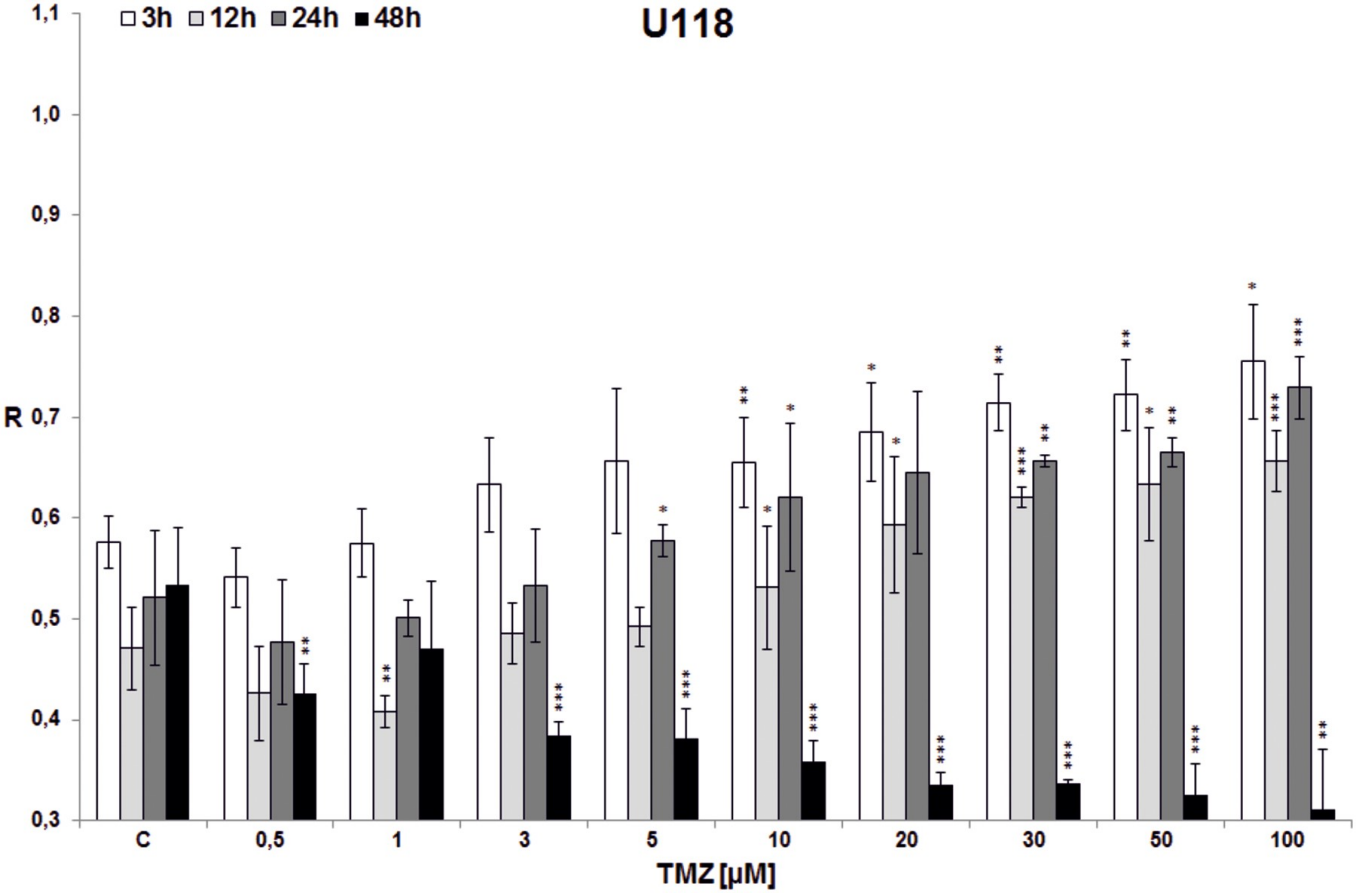
The effect of TMZ on total DNA methylation of U118 cell line. The analysis was performed after 3 (white bars), 12 (light grey bars), 24 (dark grey bars) and 48 (black bars) hrs of incubation in a given TMZ concentration (0.5–100 μM). Control cells (C) were treated with DMSO only. The R values are means from three experiments ±SD. Asterisks indicate a significant difference (*p < 0,05, **p < 0,01, ***p < 0,001) from the control (DMSO) value. Significant demethylating tendency is seen with 48 hrs incubation time in all TMZ concentrations. The TMZ amount needed to reach hypermethylating effect depends on incubation time and is within the range 1–10 μM.

**Fig 6:**
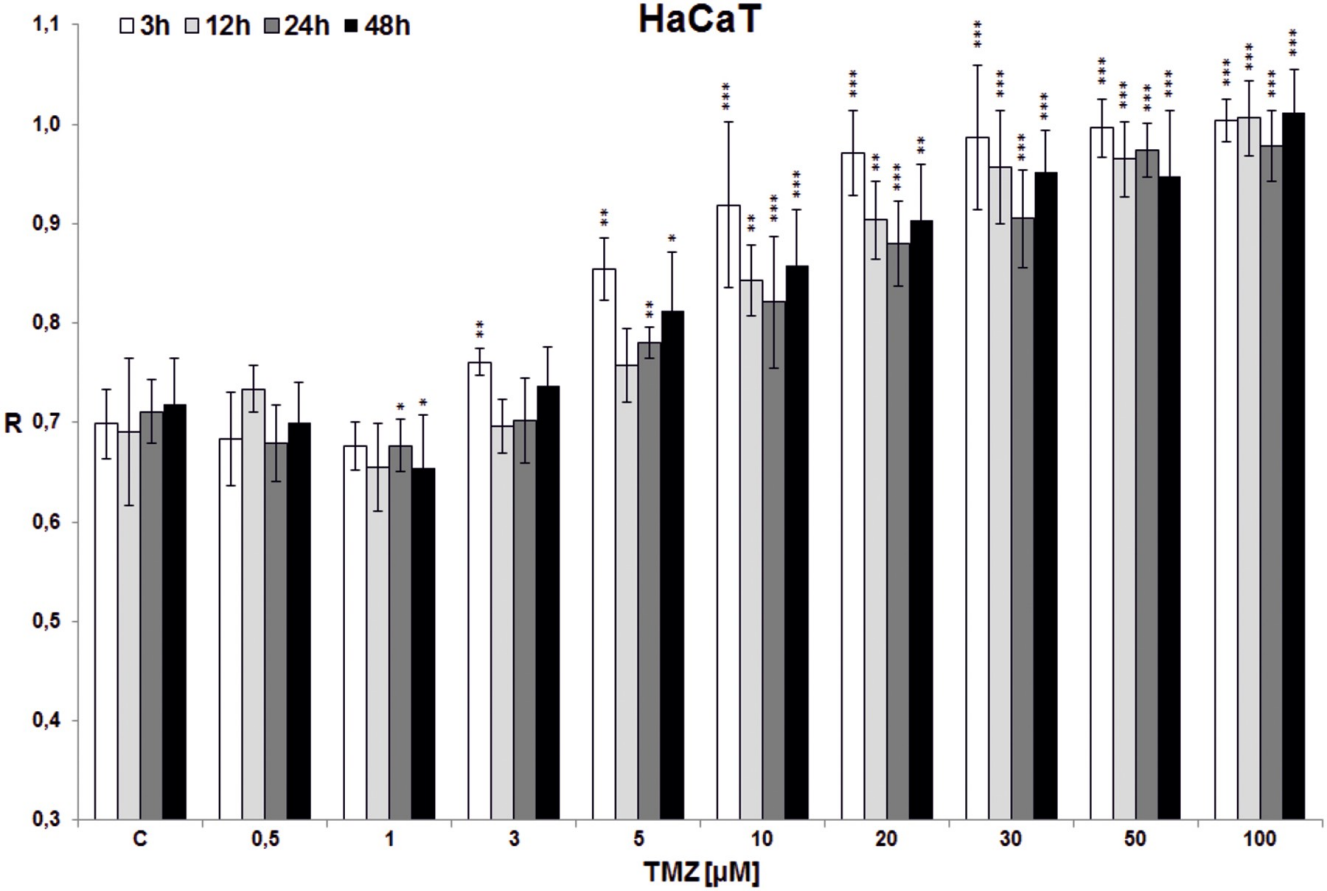
The effect of TMZ on total DNA methylation of HaCaT cell line. The analysis was performed after 3 (white bars), 12 (light grey bars), 24 (dark grey bars) and 48 (black bars) hrs of incubation in a given TMZ concentration (0.5–100 μM). Control cells (C) were treated with DMSO only. The R values are means from three experiments ±SD. Asterisks indicate a significant difference (*p < 0,05, **p < 0,01, ***p < 0,001) from the control (DMSO) value. In all incubation times the hypermethylation effect is seen over TMZ concentration of 5 μM. No hypomethylation in lower concentrations was detected.

In the first experiment total DNA methylation in cell lines (T98G, U138, U118, HaCaT) was estimated after treatment with single doses of TMZ (0.5, 1, 3, 5, 10, 20, 30, 50, 100 μM) and incubation for 3, 12, 24 and 48 hrs (Figs 3–6). Analyzing the time factor (in every set of bars of the same TMZ concentration) in glioblastoma cell lines one can clearly see DNA demethylation in all glioblastoma cell lines increased with prolonging time, and evident extreme at 48 hrs. That effect is little influenced by increasing TMZ dose to a degree depending on the line (more in U118 and T98G). Taking into account the concentration factor we observe initial DNA hypomethylation (in comparison to control) in glioblastoma cell lines in concentration range 0.5–10 μM (depending on the cell line, p values are presented in S1 Table). That effect is particularly interesting because that range covers the concentrations that are most probably reached in the brain during TMZ treatment. After reaching the concentration of 10 μM (3 μM in case of U118) dose dependent DNA hypermethylation is observed. The dependence on dose increase in all cell lines was statistically significant (***p<0.001), whereas dependence on time was observed at significant level up from doses: 3*/5**/10***μM in T98G (Fig 3), 1***μM in U138 (Fig 4), 0.5*/1**/3***μM in U118 (Fig 5), and only for TMZ 10*μM in HaCaT (Fig 6). Both phenomena were not seen in HaCaT cell line, where in general hypermethylation is detected. The results of the first experiment show that therapeutically achieved TMZ concentrations can be too low to result in hypermethylation, which is regarded as a prognostically better state [30].

**Fig 7:**
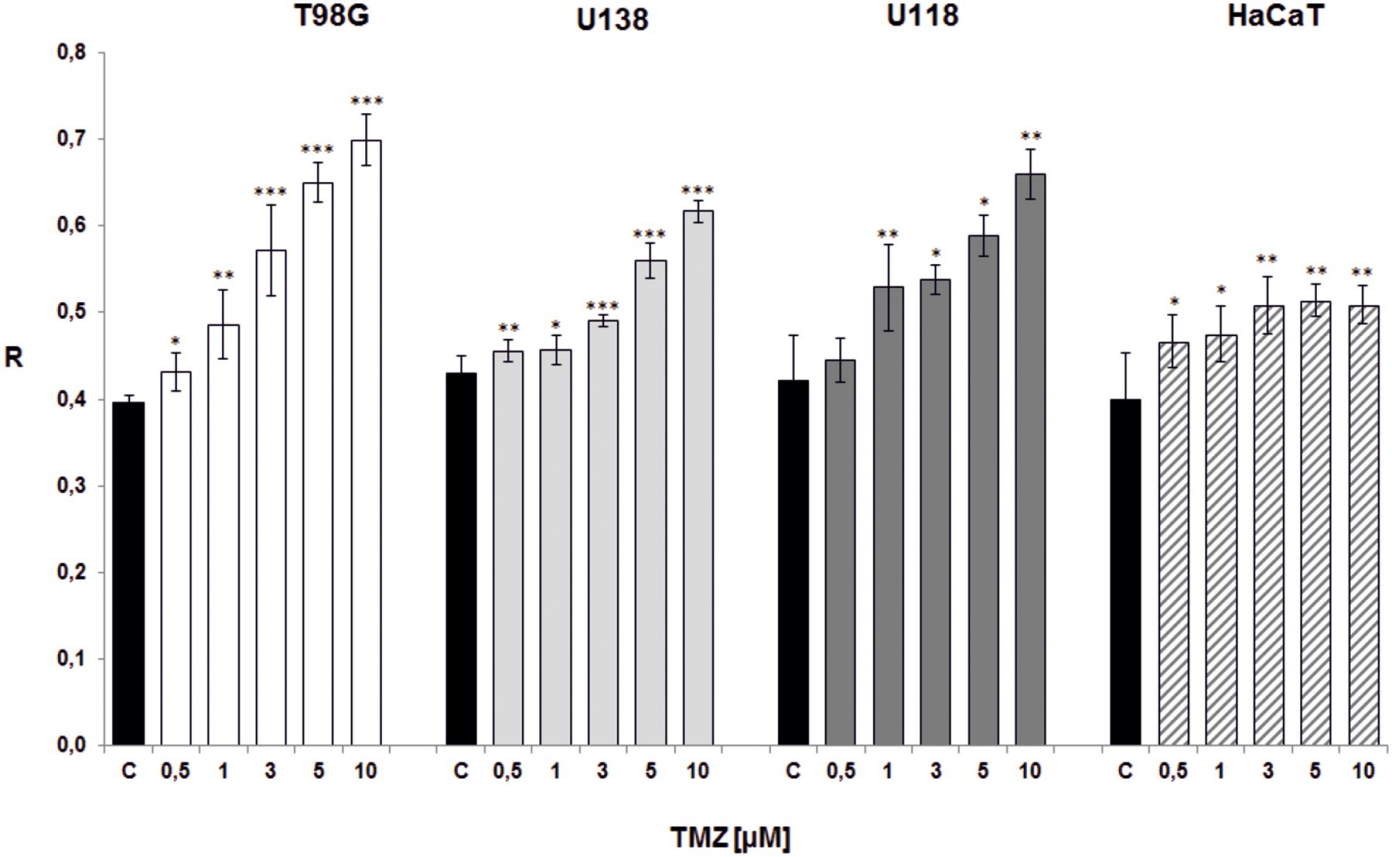
The effect of repetitive doses of TMZ on total DNA methylation. Total amount of m^5^C in DNA (R) changes during Stupp-mimicking TMZ-therapy. TMZ was administered to glioblastoma (T98G –white bars, U138 –light grey bars, U118 –dark grey bars) and keratinocyte (HaCaT–diagonally striped bars) cell lines at 0.5, 1, 3, 5, 10 μM concentrations for 5 days. Each day cells were washed with PBS and placed in fresh medium with indicated TMZ concentrations. Control (C) cells were treated with DMSO only. The total DNA methylation was estimated after 5^th^ day of treatment. The R values are means from three experiments ±SD. Asterisks indicate a significant difference (*p < 0,05, **p < 0,01, ***p < 0,001) from the control (DMSO) value.

The next experiment was intended to partially mimic the Stupp scheme for adjuvant TMZ therapy [1], and therefore to explore the effect of repetitive TMZ doses on DNA methylation. TMZ was administered to cell lines (T98G, U138, U118, HaCaT) at 0.5, 1, 3, 5, 10 μM concentrations for 5 days, wherein after 24, 48, 72 and 96 hrs cells were washed with PBS and placed in fresh medium with indicated TMZ concentrations. The total DNA methylation was estimated after 5^th^ day of treatment (Fig 7). The general TMZ dose-dependent hypermethylation is observed. Even the lowest concentration (0.5 μM) resulted with the increase of DNA methylation. That is not the case with HaCaT cell line, where rise in R level is scarce, and not clearly related to TMZ dose. One can see that repeated dosing of TMZ (every day) allows bypassing the hypomethylation effect that occurred after single therapeutically achieved TMZ doses in previous experiment (Figs 3–6). The results (S2 Table) are statistically significant for all cell lines (***p<0.001).

**Fig 8:**
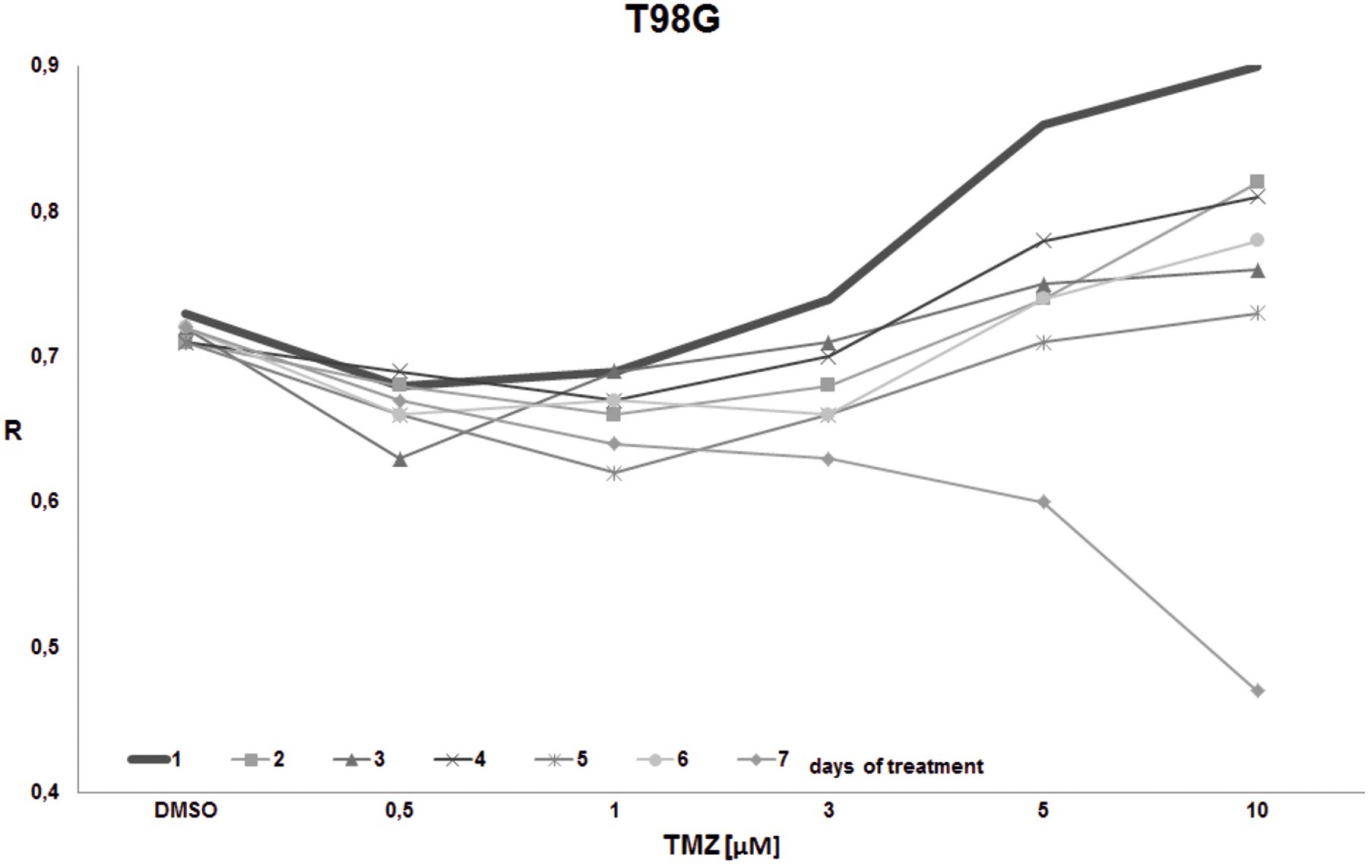
The impact of TMZ exposure period on DNA methylation in T98G cell line. T98G cell culture was treated with the given single dose of TMZ (0.5, 1, 3, 5, 10 μM) and left for 1–7 days. The global DNA methylation was estimated after 1^st^, 2^nd^, 3^rd^, 4^th^, 5^th^, and 7^th^ day of incubation. The highest methylation (R) is seen on day 1 in all concentrations. In concentrations over 3 μM we observe increasing DNA methylation. On day 7 a hypomethylating trend is observed potentiated by increasing concentration.

**Fig 9:**
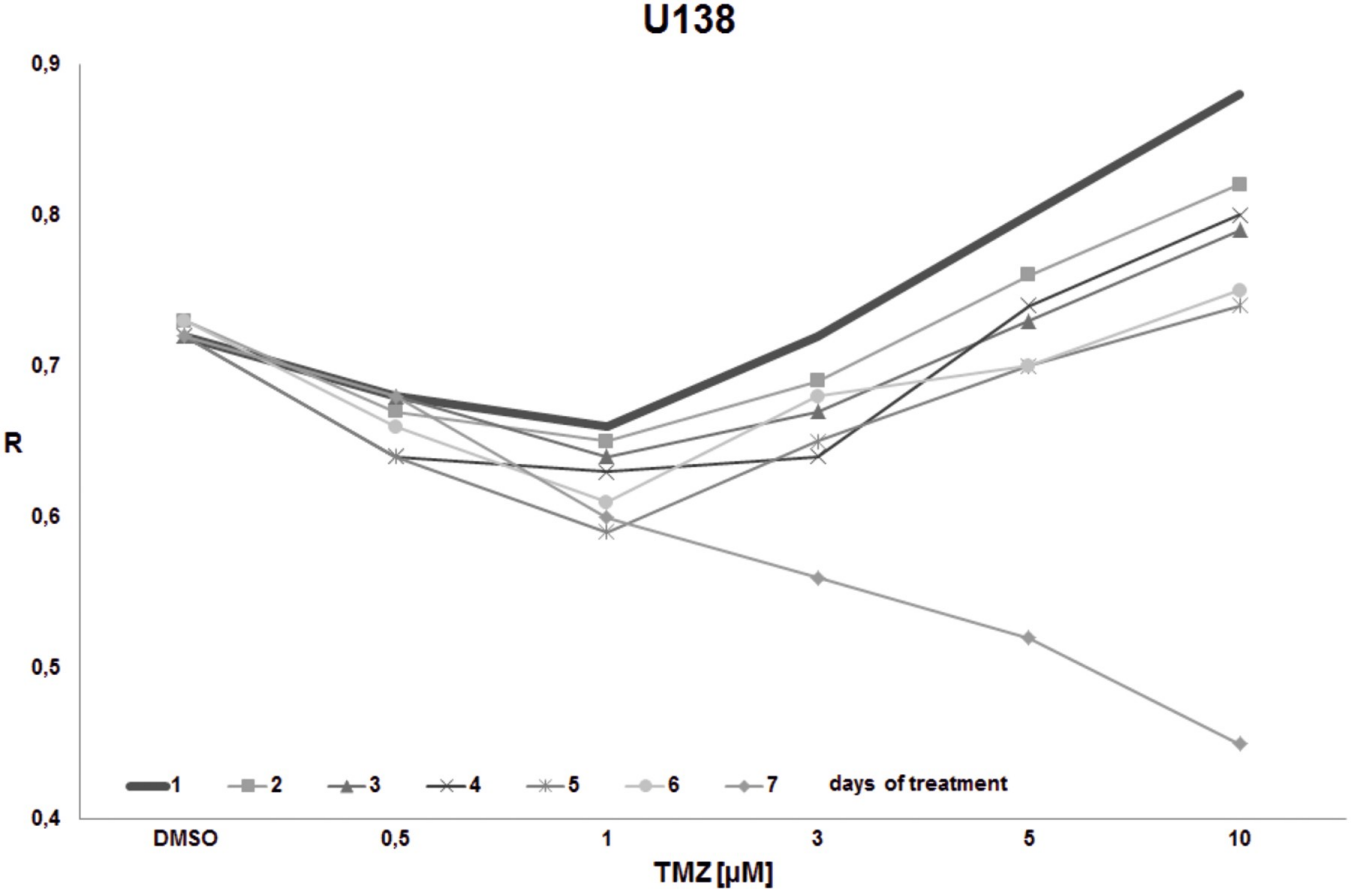
The impact of TMZ exposure period on DNA methylation in U138 cell line. U138 cell culture was treated with the given single dose of TMZ (0.5, 1, 3, 5, 10 μM) and left for 1–7 days. The global DNA methylation was estimated after 1^st^, 2^nd^, 3^rd^, 4^th^, 5^th^, and 7^th^ day of incubation. The hypomethylation at prolonged time (7 days) is clearly seen. The hypermethylation is achieved in shorter incubation times in concentrations over 5 μM.

**Fig 10:**
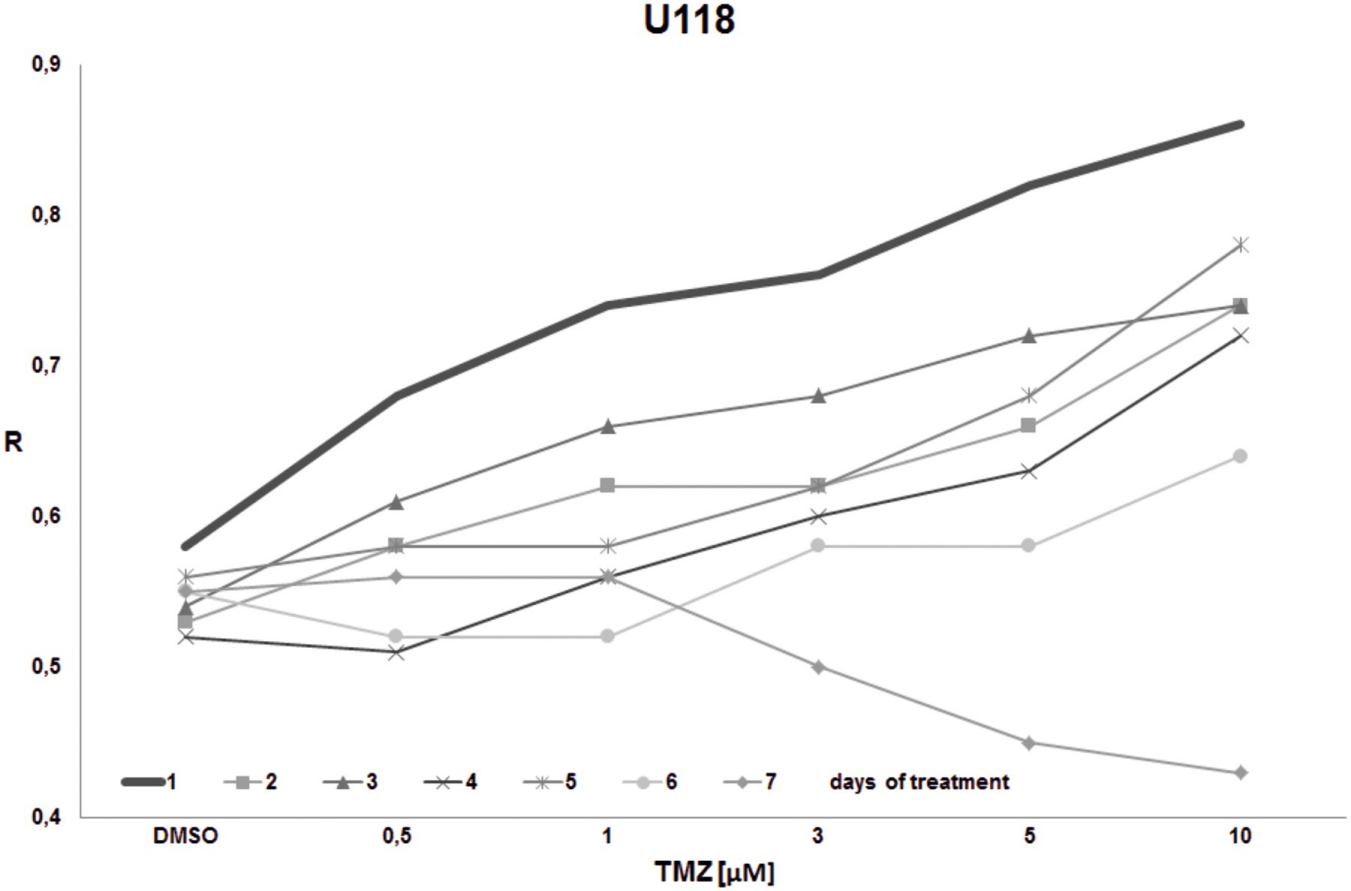
The impact of TMZ exposure period on DNA methylation in U118 cell line. U118 cell culture was treated with the given single dose of TMZ (0.5, 1, 3, 5, 10 μM) and left for 1–7 days. The global DNA methylation was estimated after 1^st^, 2^nd^, 3^rd^, 4^th^, 5^th^, and 7^th^ day of incubation. A general hypermethylation is observed, with no initial hypomethylating effect in lower concentration range. Hypomethylation after 7 days starts with concentrations over 1 μM.

**Fig 11:**
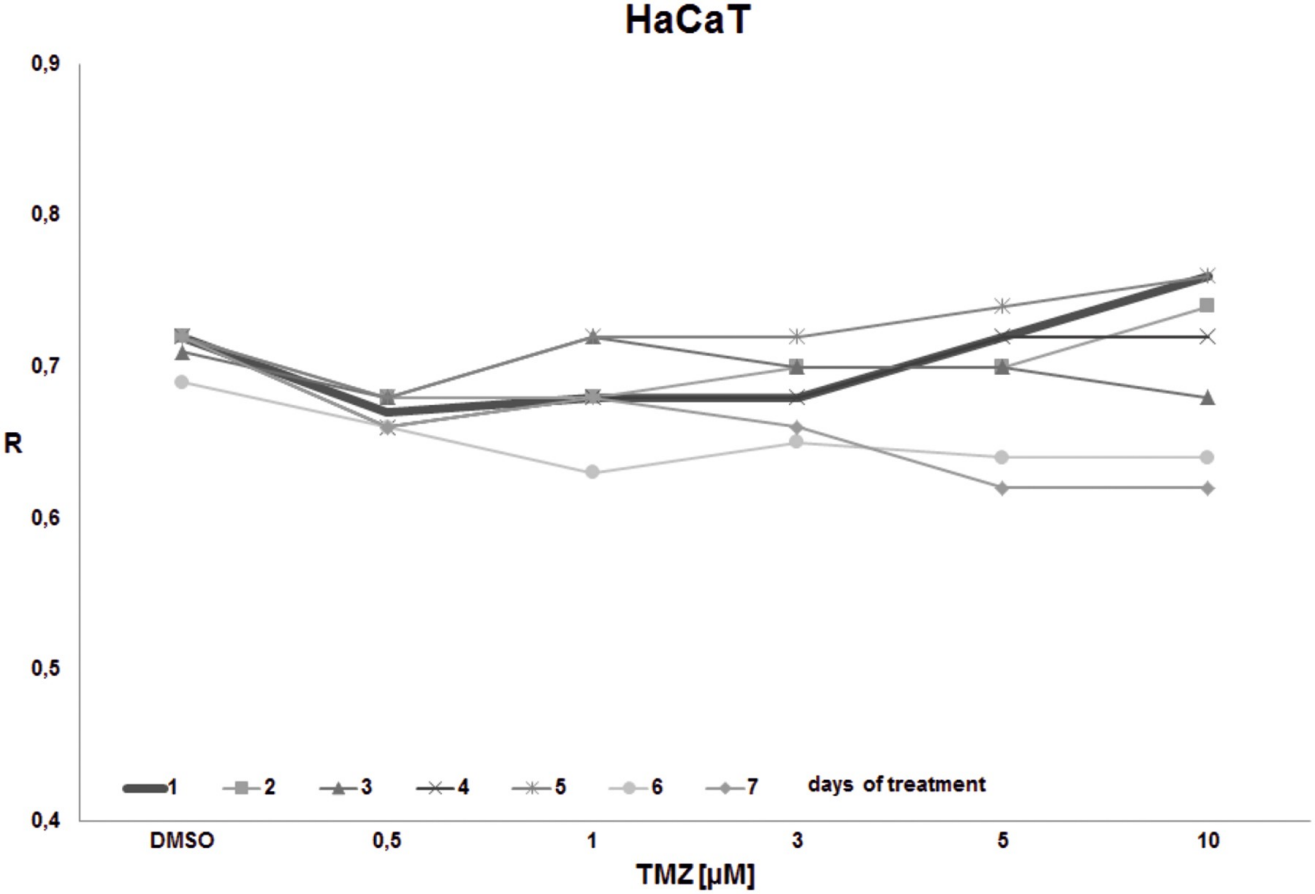
The impact of TMZ exposure period on DNA methylation in HaCaT cell line. HaCaT cell culture was treated with the given single dose of TMZ (0.5, 1, 3, 5, 10 μM) and left for 1–7 days. The global DNA methylation was estimated after 1^st^, 2^nd^, 3^rd^, 4^th^, 5^th^, and 7^th^ day of incubation. No significant change in DNA methylation is observed during time.

The goal of the final experiment was to evaluate the time effect on total DNA methylation during prolonged TMZ treatment within concentrations reachable in the brain interstitium. Cell cultures (T98G, U138, U118, HaCaT) were treated with the single dose of TMZ (0.5, 1, 3, 5, 10 μM) and were left for 1–7 days. The global DNA methylation was estimated after 1^st^, 2^nd^, 3^rd^, 4^th^, 5^th^, 6^th^, and 7^th^ day of incubation (Figs 8–11). For glioblastoma cell lines we observed general hypomethylation (when compared with day 1) tendency during time with some stabilization of R level between days 2–6, and clear drop down at day 7. The hypomethylating effect is overcome with increasing TMZ dose. In line U118 (Fig 10) all TMZ concentrations induced hypermethylation over control condition (DMSO treatment), while in T98G (Fig 8) and U138 (Fig 9) that was the case only in highest concentrations (5 and 10 μM). That effect lasted until day 6 at maximum. At day 7 general loss of DNA methylation below the control level was observed. The results of that experiment suggest time limitation of pause in chemotherapy to 4–5 days (depending on the line). The effect of time and initial TMZ dose on HaCaT (Fig 11) culture is scarce, the R values change in a very small range. The numeric results of that experiment are presented in S3 Table.

## Discussion

The aim of our work was to evaluate the effects of temozolomide action on the total contents of m^5^C in DNA of glioblastoma cell lines in conditions mimicking clinical treatment situations. The choice of the research area was driven by the fact that epigenetic modifications, particularly DNA methylation, are very sensitive to environmental (chemotherapy) changes and react faster than genetic ones, therefore that level of observation seems to be most promising in such an etiologically complex phenomenon as cancer [31,32]. In cancer, global hypomethylation and local hypermethylation are observed [33]. Global hypomethylation is regarded as a result of unspecific, broad oxidative DNA damage (all bases including m^5^C), which is associated with cancer formation and progression by many different pathways, whereas local hypermethylation is regarded as a carcinogenic event through repression of promoters of tumor suppressor genes, thereby facilitating cancer formation [34–37]. Nevertheless, globally decreased DNA methylation, which appears to correlate with carcinogenesis, is an adverse phenomenon in cancer treatment [35,37].

Decreasing amount of m^5^C in DNA of cells treated TMZ can be explained straightforward by oxidative removal of methyl group from m^5^C (demethylation) [38,39] or by demethylating activity of DNA Methyltransferases (DNMTs) in redox state, which can be induced by cancer chemotherapeutics [40,41]. Although there are data that the oxidation of 5-methylcytosine to 5-hydroxymethylcytosine can be done with oxo-reductases (TET enzymes), the mechanism of cellular DNA damage by induction of reactive oxygen species (ROS) is also plausible, The proof for that is the synthesis and presence of 8-oxo-deoxyguanosine in DNA. Newly published data provide evidence, that also in the case of loss of TET function, general DNA demethylation occurs [42]. Recently we showed fluctuations in m^5^C level in DNA and parallel 8-oxo-guanosine synthesis, which is the product of hydroxyl radical reaction with guanosine [25]. Therefore, a general conclusion is that TMZ induced genomic demethylation (hypomethylation) is a result of global non-specific oxidative stress and damage of DNA components. The level of hypomethylation depends on time and dose of treatment.

5-methylcytosine is a product of enzymatic reaction catalyzed by DNA Methyltransferases, where the only methyl donor is SAM (S-adenosyl methionine) [24]. Neither TMZ, nor TMZ degradation products are the substrates for DNMT. Therefore, the only mechanism through which a drug can increase m^5^C contents can be through the induction of DNMTs activation. The induction of DNA hypermethylation by drugs, hormones, and other biological compounds was already observed [43–45].

In our experiments we showed TMZ dose-dependent total DNA hypo- and hypermethylation, as well as repeating-dose-dependent hypermethylation. That can argue with the fact that several drugs that inhibit DNA methylation have been studied for the reactivation of tumor suppressor genes (from silencing with promoter hypermethylation) and repression of cancer cell growth [46], and also hypomethylation is regarded as an event helping with the chemotherapy effect. The problem is, the drugs don’t act selectively on hypermethylated promoters only, but on the entire DNA, resulting in global DNA hypomethylation, the hallmark of cancer, aging, and other pathologic processes [29,33,37,47,48], as well as result with mutations in epigenetic enzymes (e.g. DNMTs), that are important for response to hypomethylating treatment [49]. Moreover, DNA damage response that is activated after treatment with radiation and different classes of chemotherapeutic drugs can result both in hypersensitivity or resistance of tumors to therapy and can be exploited for improvement of cancer treatment [50]. The concept of hypomethylation as the goal of treatment has to be probably reevaluated, especially when some results show better survival in globally hypermethylated gliomas [30]. What was also observed, even if *MGMT* promoter methylation was stable, *LINE1* (global methylation surrogate) methylation status was not, resulting with decreased overall survival and GBMs post-treatment evolution with newly reprogrammed epigenetic status [51].

Besides our previous study [17] currently there are no data exploring the subject on total DNA methylation changes during TMZ therapy. In a study analyzing the impact of temozolomide in concentrations 0–200 μM during 24–72 hrs on T98G cell line Jakubowicz-Gil et al. showed that significant level of apoptosis is observed over TMZ concentration of 50 μM. That effect was potentiated with increasing incubation time, and accompanied in higher concentrations with necrosis [52]. That is in concordant with our previous results [17], and supports the results of the present study–with no significant impact on cell apoptosis, necrosis, and autophagy in the concentrations under 50 μM, the total DNA hypomethylation is observed. Similar results were showed on U118 cell line [53].

In the experiment partially mimicking the Stupp scheme (only chemotherapy factor) we observed dose-dependent increase of total DNA methylation (m^5^C), however never up to the level previously estimated to that of less malignant tumors or healthy individuals [29]. Exploring the prolonged time effect we showed that any hypermethylating treatment effect of TMZ in our experimental cell lines lasted over 5–6 days after drug intake, then having a definite demethylating trend. In the last decades a new concept in TMZ administration emerged–metronomic chemotherapy–involving low doses administered on a frequent schedule, without taking a prolonged break. Such approach appeared to be effective for primary [54] and recurrent glioblastoma [55–57]. Also prolonged (> 6 cycles) standard TMZ therapy was proven to be successful [58,59]. There are data showing that this result does not depend on *MGMT* promoter methylation [55,60], but other indicate such connection [8]. However, there are also trials that present no significant difference in overall and progression free survival after implementation of a dose-dense protocol for primary glioblastoma [60]. The positive therapeutic effect of dose-dense treatment was shown on animal models [61], however the best results were achieved for daily doses approximately 4x higher (recalculated for human conditions) than those given in a standard TMZ therapy in humans [62,63].

The question concerning a real TMZ concentration achieved in the target tissue (brain) is only partially answered [19,21–23]. The doses we tested in our experiments (0.5–10 μM), being the result of previous studies measuring real TMZ contents in the brain [19,22–23], showed DNA hypomethylation tendency compared to control. That effect was only overcome by repetitive TMZ doses. Even if not analyzed on the epigenetic background before, that effect (inadequate drug amount in target tissue) can be the reason for progression of the disease / chemotherapy resistance.

Many strategies are taken to increase the temozolomide concentration in the CNS, overcoming the boundaries of BBB [64–68]. What was shown earlier, simple implementation of radiotherapy, which is the damaging factor of BBB [69], improves the efficacy of TMZ [70]. That clinical action supports our results that increased TMZ dose results in total hypermethylation [present work, 17], and on clinical level effects in longer progression free and overall survival, as well as less general drug toxicity (because of better penetration of the given dose without the need to increase the intake).

## Conclusions

We observed dose-dependent DNA hypermethylation in glioblastoma cell lines and lack of that effect in non-cancerous cell line. A high TMZ concentration induced a significant increase of m^5^C contents in DNA even in the short time. Therefore an increase of DNA methylation, resulting in genes’ silencing and their expression down regulation, can be the reason for better glioblastoma patients’ survival. However, in the range of concentrations achieved in target tissue during standard treatment, loss of total DNA methylation is observed, what can explain the resistance to TMZ therapy and tumor recurrence. That effect is somehow overcome by repeated TMZ doses in short periods of time.

Our results show the epigenetic response to the therapeutic doses and regimens of temozolomide chemotherapy in glioblastoma. They provide a new insight into the molecular background of the treatment effects, and possible therapy modifications.

## Supporting information

S1 Table Results of total 5-methylcytosine (m^5^C) contents (R) analysis in DNA of glioblastoma (U138, T98G, U118) and HaCaT cell lines after 3, 12, 24 and 48 hours (T, time) of incubation in a given TMZ concentration [μM]. Numeric data (R) are followed with SD and p values (one-tailed *t*-test, comparison with the control–DMSO, *p < 0,05, **p < 0,01, ***p < 0,001).
(PDF)

S2 Table Results of total 5-methylcytosine (m^5^C) contents (R) analysis in DNA of glioblastoma (U138, T98G, U118) and HaCaT cell lines subject to 5-day TMZ administration scheme. Glioblastoma (U138, T98G, U118) and HaCaT cell lines were treated for 5 days with daily repeated TMZ doses [μM]. After 24, 48, 72 and 96 hrs cells were washed with PBS and placed in fresh medium with indicated TMZ concentrations. The total DNA methylation was estimated after 5^th^ day of treatment. Numeric data (R) are followed with SD and p value (one-tailed t-test, comparison with the control–DMSO, *p < 0,05, **p < 0,01, ***p < 0,001).
(PDF)

S3 Table Total 5-methylcytosine (m^5^C) contents (R) in DNA of glioblastoma (U138, T98G, U118) and HaCaT cell lines after 1–7 days (T, time) of incubation with a given single dose of TMZ [μM]. The total DNA methylation was estimated after 1^st^, 2^nd^, 3^rd^, 4^th^, 5^th^, 6^th^, and 7^th^ day of incubation. Numeric data (R) are followed with SD and p values (one-tailed *t*-test, comparison with the control–DMSO, *p < 0,05, **p < 0,01, ***p < 0,001).
(PDF)
