# Supplementary material for: Revealing the epigenetic effect of temozolomide on glioblastoma cell lines in therapeutic conditions
Source: PLoS One. 2020 Feb 26;15(2):e0229534. doi: 10.1371/journal.pone.0229534 (PMC7043761; doi:10.1371/journal.pone.0229534)
Supplement: S1 Table — Numeric data (R) are followed with SD and p values (one-tailed t-test, comparison with the control–DMSO, *p < 0,05, **p < 0,01, ***p < 0,001). (PDF) [file pone.0229534.s001.pdf]

S1 Table. Results of total 5-methylcytosine (m^5C) contents (R) analysis in DNA of glioblastoma (U138, T98G, U118) and HaCaT cell lines after 3, 12, 24 and 48 hours (T, time) of incubation in a given TMZ concentration [µM].

Numeric data (R) are followed with SD and p values (one-tailed t-test, comparison with the control – DMSO, *p < 0,05, **p < 0,01, ***p < 0,001).

TMZ	T	T98G			U138			U118			HaCaT		
[µM]	[h]	R	SD	p	R	SD	p	R	SD	p	R	SD	p
0 (DMSO)	3	0,722	0,080		0,750	0,056		0,576	0,026		0,699	0,035	
0,5	3	0,632	0,014	**	0,634	0,028	***	0,541	0,029	p>0,05	0,683	0,047	p>0,05
1,0	3	0,637	0,021	*	0,657	0,039	***	0,575	0,034	p>0,05	0,676	0,024	p>0,05
3,0	3	0,620	0,016	**	0,683	0,043	**	0,633	0,047	p>0,05	0,761	0,013	**
5,0	3	0,717	0,053	p>0,05	0,736	0,061	p>0,05	0,656	0,072	p>0,05	0,854	0,031	**
10	3	0,751	0,068	p>0,05	0,781	0,037	p>0,05	0,655	0,045	**	0,919	0,083	***
20	3	0,797	0,040	*	0,816	0,015	**	0,685	0,049	*	0,971	0,043	***
30	3	0,847	0,041	**	0,818	0,043	**	0,714	0,028	**	0,987	0,073	***
50	3	0,894	0,050	***	0,825	0,052	p>0,05	0,722	0,035	**	0,996	0,029	***
100	3	0,888	0,047	***	0,837	0,016	***	0,755	0,057	*	1,004	0,021	***
0 (DMSO)	12	0,747	0,061		0,727	0,061		0,471	0,041		0,691	0,074	
0,5	12	0,599	0,060	*	0,562	0,037	***	0,426	0,046	p>0,05	0,734	0,024	p>0,05
1,0	12	0,631	0,022	***	0,575	0,077	*	0,408	0,016	**	0,655	0,044	p>0,05
3,0	12	0,669	0,032	*	0,599	0,041	**	0,485	0,030	p>0,05	0,696	0,027	p>0,05
5,0	12	0,683	0,012	*	0,682	0,014	*	0,492	0,020	p>0,05	0,757	0,037	p>0,05
10	12	0,738	0,039	p>0,05	0,707	0,044	p>0,05	0,531	0,061	*	0,843	0,036	**
20	12	0,768	0,024	p>0,05	0,769	0,012	p>0,05	0,593	0,067	*	0,904	0,039	**
30	12	0,815	0,054	p>0,05	0,777	0,016	*	0,620	0,010	***	0,957	0,057	***

50	12	0,846	0,038	**	0,781	0,042	p>0,05	0,633	0,056	*	0,965	0,038	***
100	12	0,847	0,032	**	0,772	0,013	*	0,656	0,030	***	1,006	0,038	***
0 (DMSO)	24	0,720	0,050		0,718	0,047		0,521	0,067		0,711	0,032	
0,5	24	0,603	0,051	**	0,578	0,028	***	0,477	0,062	p>0,05	0,679	0,038	p>0,05
1,0	24	0,587	0,040	**	0,551	0,042	***	0,501	0,018	p>0,05	0,677	0,026	*
3,0	24	0,584	0,028	***	0,588	0,025	***	0,533	0,056	p>0,05	0,702	0,042	p>0,05
5,0	24	0,631	0,016	***	0,607	0,041	***	0,577	0,016	*	0,780	0,016	**
10	24	0,671	0,041	*	0,685	0,059	p>0,05	0,620	0,073	*	0,821	0,066	***
20	24	0,708	0,058	p>0,05	0,699	0,052	p>0,05	0,645	0,081	p>0,05	0,880	0,043	***
30	24	0,726	0,031	p>0,05	0,713	0,037	p>0,05	0,656	0,006	**	0,905	0,049	***
50	24	0,768	0,018	*	0,714	0,026	p>0,05	0,665	0,015	**	0,974	0,027	***
100	24	0,777	0,053	p>0,05	0,732	0,021	p>0,05	0,729	0,031	***	0,978	0,036	***
0 (DMSO)	48	0,750	0,042		0,730	0,071		0,533	0,058		0,718	0,046	
0,5	48	0,597	0,011	***	0,580	0,025	***	0,426	0,030	**	0,700	0,040	p>0,05
1,0	48	0,585	0,025	***	0,545	0,022	***	0,471	0,066	p>0,05	0,655	0,053	*
3,0	48	0,582	0,047	**	0,576	0,045	***	0,385	0,013	***	0,737	0,039	p>0,05
5,0	48	0,594	0,014	***	0,574	0,028	***	0,381	0,030	***	0,812	0,059	*
10	48	0,591	0,054	***	0,614	0,038	**	0,359	0,020	***	0,858	0,056	***
20	48	0,584	0,008	***	0,640	0,026	**	0,335	0,013	***	0,904	0,056	**
30	48	0,591	0,038	**	0,653	0,053	*	0,337	0,004	***	0,952	0,042	***
50	48	0,601	0,011	***	0,653	0,032	*	0,325	0,032	***	0,948	0,066	***
100	48	0,601	0,041	***	0,669	0,037	*	0,311	0,060	**	1,012	0,043	***
