# Supplementary material for: Revealing the epigenetic effect of temozolomide on glioblastoma cell lines in therapeutic conditions
Source: PLoS One. 2020 Feb 26;15(2):e0229534. doi: 10.1371/journal.pone.0229534 (PMC7043761; doi:10.1371/journal.pone.0229534)
Supplement: S2 Table — Glioblastoma (U138, T98G, U118) and HaCaT cell lines were treated for 5 days with daily repeated TMZ doses [μM]. After 24, 48, 72 and 96 hrs cells were washed with PBS and placed in fresh medium with indicated TMZ concentrations. The total DNA methylation was estimated after 5th day of treatment. Numeric data (R) are followed with SD and p value (one-tailed t-test, comparison with the control–DMSO, *p < 0,05, **p < 0,01, ***p < 0,001). (PDF) [file pone.0229534.s002.pdf]

**S2 Table. Results of total 5-methylcytosine ($m^5C$) contents (R) analysis in DNA of glioblastoma (U138, T98G, U118) and HaCaT cell lines subject to 5-day TMZ administration scheme.**

Glioblastoma (U138, T98G, U118) and HaCaT cell lines were treated for 5 days with daily repeated TMZ doses [µM]. After 24, 48, 72 and 96 hrs cells were washed with PBS and placed in fresh medium with indicated TMZ concentrations. The total DNA methylation was estimated after 5th day of treatment. Numeric data (R) are followed with SD and p value (one-tailed t-test, comparison with the control – DMSO, *p < 0,05, **p < 0,01, ***p < 0,001).

| TMZ | T | T98G | | | U138 | | | U118 | | | HaCaT | | |
|---|---|---|---|---|---|---|---|---|---|---|---|---|---|
| [µM] | [days] | R | SD | p | R | SD | p | R | SD | p | R | SD | p |
| 0 (DMSO) | 5 | 0,396 | 0,008 | | 0,428 | 0,020 | | 0,421 | 0,052 | | 0,400 | 0,053 | |
| 0,5 | 5 | 0,432 | 0,022 | * | 0,456 | 0,012 | ** | 0,445 | 0,025 | p>0,05 | 0,467 | 0,030 | * |
| 1,0 | 5 | 0,486 | 0,040 | ** | 0,457 | 0,017 | * | 0,529 | 0,050 | ** | 0,475 | 0,032 | * |
| 3,0 | 5 | 0,572 | 0,052 | *** | 0,491 | 0,007 | *** | 0,538 | 0,017 | * | 0,509 | 0,033 | ** |
| 5,0 | 5 | 0,650 | 0,023 | *** | 0,560 | 0,020 | *** | 0,589 | 0,024 | * | 0,514 | 0,019 | ** |
| 10 | 5 | 0,699 | 0,030 | *** | 0,617 | 0,013 | *** | 0,660 | 0,029 | ** | 0,509 | 0,022 | ** |
