# Supplementary material for: Revealing the epigenetic effect of temozolomide on glioblastoma cell lines in therapeutic conditions
Source: PLoS One. 2020 Feb 26;15(2):e0229534. doi: 10.1371/journal.pone.0229534 (PMC7043761; doi:10.1371/journal.pone.0229534)
Supplement: S3 Table — The total DNA methylation was estimated after 1st, 2nd, 3rd, 4th, 5th, 6th, and 7th day of incubation. Numeric data (R) are followed with SD and p values (one-tailed t-test, comparison with the control–DMSO, *p < 0,05, **p < 0,01, ***p < 0,001). (PDF) [file pone.0229534.s003.pdf]

S3 Table. Total 5-methylcytosine (m^5C) contents (R) in DNA of glioblastoma (U138, T98G, U118) and HaCaT cell lines after 1-7 days (T, time) of incubation with a given single dose of TMZ [µM].

The total DNA methylation was estimated after 1st, 2nd, 3rd, 4th, 5th, 6th, and 7th day of incubation. Numeric data (R) are followed with SD and p values (one-tailed t-test, *p < 0,05, **p < 0,01, ***p < 0,001).

TMZ	T	T98G			U138			U118			HaCaT		
[µM]	[days]	R	SD	p	R	SD	p	R	SD	p	R	SD	p
0 (DMSO)	1	0,725	0,013		0,722	0,051		0,575	0,014		0,722	0,033	
0,5	1	0,676	0,016	*	0,678	0,023	p>0,05	0,676	0,045	*	0,666	0,039	*
1,0	1	0,694	0,058	p>0,05	0,661	0,038	p>0,05	0,738	0,041	**	0,681	0,072	p>0,05
3,0	1	0,737	0,051	p>0,05	0,721	0,031	p>0,05	0,764	0,040	**	0,681	0,067	p>0,05
5,0	1	0,861	0,059	**	0,801	0,027	*	0,816	0,024	***	0,720	0,053	p>0,05
10	1	0,897	0,038	***	0,877	0,052	**	0,863	0,036	***	0,757	0,044	p>0,05
0 (DMSO)	2	0,714	0,044		0,731	0,014		0,526	0,021		0,722	0,034	
0,5	2	0,684	0,023	p>0,05	0,672	0,053	p>0,05	0,575	0,034	p>0,05	0,681	0,028	p>0,05
1,0	2	0,660	0,031	p>0,05	0,648	0,048	*	0,622	0,050	*	0,685	0,063	p>0,05
3,0	2	0,679	0,067	p>0,05	0,692	0,026	*	0,619	0,058	p>0,05	0,695	0,041	p>0,05
5,0	2	0,743	0,027	p>0,05	0,756	0,066	p>0,05	0,655	0,027	**	0,695	0,056	p>0,05
10	2	0,820	0,053	*	0,816	0,047	**	0,740	0,060	**	0,741	0,023	p>0,05
0 (DMSO)	3	0,715	0,028		0,717	0,043		0,538	0,019		0,706	0,029	
0,5	3	0,630	0,019	**	0,683	0,041	p>0,05	0,608	0,012	**	0,678	0,025	*
1,0	3	0,688	0,011	p>0,05	0,645	0,046	p>0,05	0,663	0,007	***	0,725	0,058	p>0,05
3,0	3	0,712	0,037	p>0,05	0,670	0,017	p>0,05	0,682	0,054	*	0,703	0,020	p>0,05
5,0	3	0,746	0,060	p>0,05	0,726	0,026	p>0,05	0,724	0,041	**	0,704	0,026	p>0,05
10	3	0,757	0,043	p>0,05	0,788	0,023	p>0,05	0,739	0,034	***	0,679	0,069	p>0,05

0 (DMSO)	4	0,711	0,028		0,724	0,029		0,521	0,011		0,724	0,009	
0,5	4	0,687	0,029	p>0,05	0,641	0,057	p>0,05	0,512	0,074	p>0,05	0,655	0,031	*
1,0	4	0,667	0,031	*	0,632	0,024	*	0,558	0,017	*	0,679	0,016	*
3,0	4	0,699	0,021	p>0,05	0,643	0,013	**	0,597	0,017	**	0,676	0,017	*
5,0	4	0,777	0,010	*	0,741	0,065	p>0,05	0,628	0,103	p>0,05	0,725	0,020	p>0,05
10	4	0,810	0,030	**	0,802	0,026	p>0,05	0,717	0,045	**	0,719	0,006	p>0,05
0 (DMSO)	5	0,708	0,046		0,719	0,071		0,563	0,030		0,721	0,041	
0,5	5	0,661	0,081	p>0,05	0,635	0,016	p>0,05	0,576	0,053	p>0,05	0,677	0,049	p>0,05
1,0	5	0,619	0,031	*	0,589	0,071	*	0,584	0,075	p>0,05	0,721	0,030	p>0,05
3,0	5	0,657	0,065	p>0,05	0,654	0,049	p>0,05	0,618	0,061	p>0,05	0,720	0,046	p>0,05
5,0	5	0,710	0,055	p>0,05	0,698	0,015	p>0,05	0,678	0,009	**	0,742	0,035	p>0,05
10	5	0,726	0,050	p>0,05	0,744	0,006	p>0,05	0,776	0,020	***	0,761	0,031	p>0,05
0 (DMSO)	6	0,719	0,032		0,731	0,027		0,549	0,041		0,693	0,033	
0,5	6	0,664	0,019	*	0,664	0,072	p>0,05	0,524	0,019	p>0,05	0,664	0,054	p>0,05
1,0	6	0,674	0,021	*	0,615	0,019	**	0,517	0,014	p>0,05	0,633	0,038	p>0,05
3,0	6	0,663	0,019	*	0,679	0,023	p>0,05	0,581	0,017	p>0,05	0,653	0,042	p>0,05
5,0	6	0,736	0,043	p>0,05	0,699	0,050	p>0,05	0,584	0,047	p>0,05	0,636	0,035	p>0,05
10	6	0,776	0,040	*	0,750	0,054	p>0,05	0,641	0,033	*	0,640	0,033	p>0,05
0 (DMSO)	7	0,718	0,030		0,725	0,041		0,550	0,015		0,717	0,039	
0,5	7	0,672	0,028	p>0,05	0,679	0,045	p>0,05	0,557	0,011	p>0,05	0,655	0,076	p>0,05
1,0	7	0,639	0,008	*	0,599	0,054	p>0,05	0,565	0,038	p>0,05	0,678	0,030	p>0,05
3,0	7	0,626	0,022	*	0,563	0,044	**	0,500	0,048	p>0,05	0,662	0,040	p>0,05
5,0	7	0,596	0,040	*	0,516	0,052	*	0,449	0,029	**	0,623	0,012	*
10	7	0,469	0,029	***	0,452	0,049	**	0,431	0,040	**	0,622	0,063	p>0,05
